# Guidelines for Preventing and Treating Vitamin D Deficiency: A 2023 Update in Poland

**DOI:** 10.3390/nu15030695

**Published:** 2023-01-30

**Authors:** Paweł Płudowski, Beata Kos-Kudła, Mieczysław Walczak, Andrzej Fal, Dorota Zozulińska-Ziółkiewicz, Piotr Sieroszewski, Jarosław Peregud-Pogorzelski, Ryszard Lauterbach, Tomasz Targowski, Andrzej Lewiński, Robert Spaczyński, Mirosław Wielgoś, Jarosław Pinkas, Teresa Jackowska, Ewa Helwich, Artur Mazur, Marek Ruchała, Arkadiusz Zygmunt, Mieczysław Szalecki, Artur Bossowski, Justyna Czech-Kowalska, Marek Wójcik, Beata Pyrżak, Michał A. Żmijewski, Paweł Abramowicz, Jerzy Konstantynowicz, Ewa Marcinowska-Suchowierska, Andrius Bleizgys, Spirydon N. Karras, William B. Grant, Carsten Carlberg, Stefan Pilz, Michael F. Holick, Waldemar Misiorowski

**Affiliations:** 1 Department of Biochemistry, Radioimmunology and Experimental Medicine, The Children’s Memorial Health Institute, 04-730 Warsaw, Poland; 2 Department of Endocrinology and Neuroendocrine Tumors, and the Department of Pathophysiology and Endocrinology in Zabrze, Medical University of Silesia, 40-952 Katowice, Poland; 3 Department of Pediatrics, Endocrinology, Diabetology, Metabolic Diseases and Cardiology of Developmental Age, Pomeranian Medical University, 70-204 Szczecin, Poland; 4 Department of Allergy, Lung Diseases and Internal Medicine of the Central Clinical Hospital, Ministry of Interior, 02-507 Warsaw, Poland; 5 Department and Clinic of Internal Diseases and Diabetology, Medical University of Poznan, 60-834 Poznan, Poland; 6 Department of Fetal Medicine and Gynecology, Medical University of Łodz, 90-419 Łodz, Poland; 7 Department of Pediatrics, Oncology and Pediatric Immunology, Pomeranian Medical University, 70-204 Szczecin, Poland; 8 Department of Neonatology, Jagiellonian University Hospital, 31-501 Krakow, Poland; 9 Department of Geriatrics, National Institute of Geriatrics, Rheumatology and Rehabilitation, 02-637 Warsaw, Poland; 10 Department of Endocrinology and Metabolic Diseases, Medical University of Łodz, 93-338 Łodz, Poland; 11 Center for Gynecology, Obstetrics and Infertility Pastelova, 60-198 Poznan, Poland; 12 Department of Obstetrics and Gynecology, Medical University of Warsaw, 02-015 Warsaw, Poland; 13 School of Public Health, The Center of Postgraduate Medical Education, 01-826 Warsaw, Poland; 14 Department of Pediatrics, Centre for Postgraduate Medical Education, 01-813 Warsaw, Poland; 15 Department of Neonatology and Neonatal Intensive Care, Institute of Mother and Child, 01-211 Warsaw, Poland; 16 Institute of Medical Sciences, Medical College of Rzeszow University, 35-310 Rzeszow, Poland; 17 Department of Endocrinology, Metabolism and Internal Medicine, Medical University of Poznan, 60-355 Poznan, Poland; 18 Department of Endocrinology and Diabetology, The Children’s Memorial Health Institute, 04-730 Warsaw, Poland, and the Collegium Medicum, Jan Kochanowski University, 25-317 Kielce, Poland; 19 Department of Pediatrics, Endocrinology, Diabetology with Cardiology Unit, Medical University of Bialystok, 15-274 Bialystok, Poland; 20 Department of Neonatology and Neonatal Intensive Care, The Children’s Memorial Health Institute, 04-730 Warsaw, Poland; 21 Department of Pediatrics and Endocrinology, Medical University of Warsaw, 02-091 Warsaw, Poland; 22 Department of Histology, Medical University of Gdansk, 80-211 Gdansk, Poland; 23 Department of Pediatrics, Rheumatology, Immunology and Metabolic Bone Diseases, Medical University of Bialystok, 15-274 Bialystok, Poland; 24 Department of Internal Medicine and Geriatric Cardiology, and the Department of Geriatrics and Gerontology, School of Public Health, The Center of Postgraduate Medical Education, 02-673 Warsaw, Poland; 25 Clinic of Internal Diseases, Family Medicine and Oncology, Institute of Clinical Medicine, Faculty of Medicine, Vilnius University, 08661 Vilnius, Lithuania; 26 Laboratory of Biological Chemistry, Medical School, Aristotle University, 55535 Thessaloniki, Greece; 27 Sunlight, Nutrition and Health Research Center, San Francisco, CA 94164, USA; 28 Institute of Animal Reproduction and Food Research, Polish Academy of Science, 10-748 Olsztyn, Poland; 29 Department of Internal Medicine, Division of Endocrinology and Diabetology, Medical University of Graz, 8036 Graz, Austria; 30 Section Endocrinology, Diabetes, Nutrition and Weight Management, Department of Medicine, Boston University School of Medicine, Boston, MA 02118, USA; 31 Department of Endocrinology, Centre of Postgraduate Medical Education, Bielanski Hospital, 01-809 Warsaw, Poland

**Keywords:** vitamin D deficiency, 25-hydroxyvitamin-D, general population guidelines, risk groups, calcifediol, cholecalciferol

## Abstract

**Introduction:** All epidemiological studies suggest that vitamin D deficiency is prevalent among the Polish general population. Since vitamin D deficiency was shown to be among the risk factors for many diseases and for all-cause mortality, concern about this problem led us to update the previous Polish recommendations. **Methods:** After reviewing the epidemiological evidence, case-control studies and randomized control trials (RCTs), a Polish multidisciplinary group formulated questions on the recommendations for prophylaxis and treatment of vitamin D deficiency both for the general population and for the risk groups of patients. The scientific evidence of pleiotropic effects of vitamin D as well as the results of panelists’ voting were reviewed and discussed. Thirty-four authors representing different areas of expertise prepared position statements. The consensus group, representing eight Polish/international medical societies and eight national specialist consultants, prepared the final Polish recommendations. **Results:** Based on networking discussions, the ranges of total serum 25-hydroxyvitamin D concentration indicating vitamin D deficiency [<20 ng/mL (<50 nmol/L)], suboptimal status [20–30 ng/mL (50–75 nmol/L)], and optimal concentration [30–50 ng/mL (75–125 nmol/L)] were confirmed. Practical guidelines for cholecalciferol (vitamin D_3_) as the first choice for prophylaxis and treatment of vitamin D deficiency were developed. Calcifediol dosing as the second choice for preventing and treating vitamin D deficiency was introduced. **Conclusions:** Improving the vitamin D status of the general population and treatment of risk groups of patients must be again announced as healthcare policy to reduce a risk of spectrum of diseases. This paper offers consensus statements on prophylaxis and treatment strategies for vitamin D deficiency in Poland.

## 1. Introduction

Vitamin D metabolism and its role in human health and disease have been studied, showing a broad spectrum of pleiotropic effects. Vitamin D from the diet or through the cutaneous synthesis as well as from supplements, over the counter drugs (OTC), or prescription drugs, is subsequently hydroxylated in the liver to 25-hydroxyvitamin D, i.e., 25(OH)D, and then in the kidneys, forming biologically active metabolite 1,25-dihydroxyvitamin D. Of note, vitamin D_2_ coming from sun dried mushrooms and UV irradiated yeast, and vitamin D_3_ originating from sun exposure and the dietary intake of oily fish, cod liver oil and supplemented foods are both metabolized in the liver to 25-hydroxyvitamin D. In fact, 25(OH)D represents either or both 25-hydroxyvitamin D_2_ and 25-hydroxyvitamin D_3_ and should be treated as the major circulating metabolite form, with a longer mean half-life of about 13–15 days, in comparison to other chemical forms. The two main pathways of degradation of both 25(OH)D and 1,25(OH)_2_D are the C23 lactone pathway and the C24 oxidation pathway. The abovementioned vitamin D metabolites are degraded by the actions of CYP24A1 (24-hydroxylase). After several steps, calcitroic acid, one of the end products of the C24 oxidation pathway, is excreted, mainly in the bile and thus in the feces.

The currently available epidemiological data support the view that vitamin D deficiency is common worldwide, including Poland [1,2,3,4,5]. However, associations between vitamin D status and global and public health have not been fully explored yet. Furthermore, most but not all published studies, reporting health risks and morbidity due to vitamin D deficiency, demonstrate good and well-balanced evidence [6,7,8,9,10]. A number of negative studies regarding vitamin D effects should also be acknowledged in the literature from the last decade [11,12,13,14,15]. Some recent data based on RCTs with vitamin D have shown contradictory results. However, conclusions from the majority of those reports, particularly using extension studies, prolonged observation time, and specific endpoints, demonstrate the beneficial effects of vitamin D in cancer prevention and the all-cause mortality rate [6,7].

In Poland, the history of vitamin D started in 1822 when Dr. Śniadecki found the relation between nutritional rickets and sun exposure in children living in big cities compared to rural areas in Poland. The first vitamin D recommendations were prepared and published in Poland in 2009, followed by the second and third in 2013 and 2018 [16,17,18]. Table 1 provides insights into those guidelines. The rationale of the present consensus paper and the updated recommendations was based on the compelling evidence and increasing amount of information on the effects of vitamin D on health in all age groups. The objective was to provide a concise and organized practice guidance for preventive supplementation and the management of the deficiency.

## 2. Methods

This document provides the consensus agreement of a joint expert panel and a working group with contributors, expert clinicians representing national medical societies, and national consultants involved due to their expertise as well as recognized researchers having a consequential and significant track record in the field of vitamin D, particularly regarding associations with major health problems, global health, epidemiology of the deficiency, and relations to human morbidity.

Agreement on established recommendations was finally achieved after extensive, balanced, and comprehensive discussions and revisions of the document, leading to a consensus on all items. On this occasion, the panel members decided to refrain from grading the strength of the recommendations, thus, no quantitative rating of the evidence was used.

An executive writing group (P.P.; W.B.G.; E.M-S.; P.A.; J.K.; M.A.Ż.; W.M.) was appointed to prepare the first draft of the manuscript, and this draft manuscript was then sent to an expert group for critical revision. The most recent evidence published following the expert group discussions was considered and included in the draft by the executive writing group. The first author was responsible for the preparation of the final version of the manuscript and for sending it to the entire expert group for final approval of the content and of the final recommendations. Following the unanimous final endorsement of the recommendations and content by all expert group members, the document was submitted for publication.

## 3. Recommendations on Vitamin D: A 2023 Update

### Outline of the General Recommendations

(1) Prevention and treatment schedules of vitamin D deficiency in Poland are based on the use of cholecalciferol or, under specific medical conditions, on the use of calcifediol. Cholecalciferol should be considered as the first choice for both prophylactic and treatment options. Calcifediol should be used as the second choice, when cholecalciferol use does not improve serum 25(OH)D concentration or an immediate increase in serum 25(OH)D is required.
(2) Prevention of vitamin D deficiency in the general population with the use of cholecalciferol should be individualized depending on age, body weight, the sun exposure of an individual, dietary habits and lifestyle.
(3) If disease-specific practice guidelines are not available, preventive treatment of vitamin D deficiency in the risk groups should be implemented according to arrangements for the general population; the maximal admissible daily doses of cholecalciferol (Table 2) for a given age group in the general population are recommended for use in the risk groups of vitamin D deficiency.
(4) In the general population with documented vitamin D deficiency, the dosing of cholecalciferol (or calcifediol) should be based on serum 25(OH)D concentration and chronological (calendar) age, and in case of cholecalciferol, additionally on body weight.
(5) In the risk groups, in case of vitamin D deficiency documented by laboratory assays, the cholecalciferol (or calcifediol) treatment and dosage adjustment should be based on 25(OH)D concentration as well as age, the nature of the underlying disease, medical therapy, and in case of cholecalciferol, additionally on body weight.
(6) Adjusting the dosing regimen to the patient’s preference and supplementing on a weekly or monthly basis may positively impact adherence.
(7) In the general population, the specific indications for 25(OH)D assay testing were not established and the screening of serum 25(OH)D in the entire population is not recommended.
(8) In the risk groups, the evaluation of vitamin D status, based on serum 25(OH)D assay, is strongly recommended.
(9) If supplementation with use of calcifediol in daily doses of 10 µg in oral solution is required for medical reasons, the first control of serum 25(OH)D is recommended within 6–8 days.

**Table 2:** Upper limits for daily cholecalciferol intake for vitamin D deficiency prophylaxis in the general population by age.

Age	Tolerable Upper Intake Level, IU/day (µg/day)
Neonates and infants aged 0–12 months	1000 (25)
Children aged 1–10 years	2000 (50)
Adolescents aged 11–18 years	4000 (100)
Adults aged 19 years and older with normal body weight	4000 (100)
Pregnant and breastfeeding women	4000 (100)
Adults aged 19 years and older with overweight or obesity	10,000 (250)

## 4. Prevention of Vitamin D Deficiency and Insufficiency: Recommendations for the General Population

### 4.1. Neonates Born at Term and Infants

(1) Age 0–6 months: 400 IU/day (10 µg/day) of cholecalciferol from first days of life, regardless of the feeding method.
(2) Age 6–12 months: 400–600 IU/day (10–15 µg/day) of cholecalciferol, depending on the daily amount of vitamin D consumed with meals.
(3) In term-born neonates and healthy infants calcifediol is not recommended.

### 4.2. Children (1–10 Years)

(1) In healthy children aged 1–3 years, supplementation should be based on cholecalciferol administration provided in a daily dose of 600 IU (15 µg/day) and, due to age-related restrictions of sunbathing, is recommended throughout the year.
(2) In healthy children aged 4–10 years sunbathing with uncovered forearms and legs for 15–30 minutes between 10 a.m. and 3 p.m. without sunscreen, starting from May until the end of September, cholecalciferol supplementation is not necessary, although still recommended and safe.
(3) If these guidelines are not fulfilled in healthy children aged 4–10 years, supplementation of cholecalciferol in dose 600–1000 IU/day (15–25 µg/day) is recommended throughout the year, based on body weight and the dietary vitamin D intake.
(4) In healthy children aged 1–10 years, calcifediol is not recommended.

### 4.3. Adolescents (11–18 Years)

(1) In healthy adolescents, cholecalciferol as the first choice of supplementation and calcifediol as the second choice should both be used for the prevention of vitamin D deficiency.
(2) In healthy adolescents, sunbathing with uncovered forearms and legs for 30–45 minutes between 10 a.m. and 3 p.m. without sunscreen, starting from May until the end of September, cholecalciferol supplementation is not necessary, although still recommended and safe.
(3) If these guidelines are not fulfilled, supplementation based on cholecalciferol in a dose of 1000–2000 IU/day (25–50 µg/day) is recommended throughout the year, based on body weight and the dietary vitamin D intake.
(4) If the above guidelines are not fulfilled, alternative prevention based on calcifediol in a daily dose of 10 µg (oral solution) is recommended throughout the year and the control assay of serum 25(OH)D should be performed 6–8 days after starting supplementation.

### 4.4. Adults (19–65 Years)

(1) In healthy adults, cholecalciferol as the first choice of supplementation and calcifediol as the second choice should be both used for the prevention of vitamin D deficiency.
(2) In healthy adults sunbathing with uncovered forearms and legs for 30–45 minutes between 10 a.m. and 3 p.m., without sunscreens starting from May until the end of September, cholecalciferol supplementation is not necessary, although still recommended and safe.
(3) If these guidelines are not fulfilled, supplementation based on cholecalciferol in a dose of 1000–2000 IU/day (25–50 µg/day) is recommended throughout the year, based on body weight and the dietary vitamin D intake.
(4) If the above guidelines are not fulfilled, alternative prevention based on calcifediol in a daily dose of 10 µg (oral solution) is recommended throughout the year and the control assay of serum 25(OH)D should be performed 6–8 days after starting supplementation.

### 4.5. Younger Seniors (>65 Years), Older Seniors (>75 Years), Oldest Old Seniors (90 Years and Older)

#### 4.5.1. Younger Seniors (>65–75 Years)

(1) In younger seniors’ cholecalciferol as the first choice of supplementation and calcifediol as the second choice should be both used for the prevention of vitamin D deficiency.
(2) Due to decreased efficacy of the skin synthesis, supplementation based on cholecalciferol in a dose of 1000–2000 IU/day (25–50 µg/day), based on body weight and the dietary vitamin D intake, is recommended throughout the year.
(3) If the above guidelines are not fulfilled, calcifediol in a daily dose of 10 µg (oral solution) as an alternative prevention is recommended throughout the year and the control assay of serum 25(OH)D should be performed 6–8 days after starting supplementation.

#### 4.5.2. Older Seniors (>75–89 Years) and the Oldest Old Seniors (90 Years and Older)

(1) In older seniors and in the oldest old seniors, cholecalciferol as the first choice of supplementation and calcifediol as the second choice should both be used for the prevention of vitamin D deficiency.
(2) Due to decreased efficacy of the skin synthesis, potential malabsorption and altered metabolism of vitamin D, cholecalciferol supplementation of 2000–4000 IU/day (50–100 µg/day), based on body weight and the dietary vitamin D intake, is recommended throughout the year;
(3) Calcifediol in a daily dose of 10 µg (oral solution) as the alternative prevention is recommended throughout the year if the above guidelines are not fulfilled and the control assay of serum 25(OH)D should be performed 6–8 days after starting supplementation.

### 4.6. Pregnancy and Lactation

(1) Women planning pregnancy should receive adequate cholecalciferol supplementation (or—if reasonable—alternatively calcifediol), the same as in the general adult population, if possible, under the control of serum 25(OH)D concentration.
(2) When pregnancy is confirmed until the end of breastfeeding, cholecalciferol supplementation should be carried out under the control of 25(OH)D concentration to achieve and maintain optimal concentrations within the ranges of >30–50 ng/mL.
(3) If the assessment of serum 25(OH)D concentration is not accessible, it is recommended to use cholecalciferol at a dose of 2000 IU/day (50 µg/day), throughout pregnancy and lactation.
(4) In some very specific medical conditions, calcifediol in a daily dose of 10 µg (oral solution) as an alternative prevention could be considered throughout a pregnancy and lactation with special medical supervision. Warning: this recommendation is out of the label of the registration indications of the drug.

### 4.7. Preterm Neonates

#### 4.7.1. Neonates Born at <32 Weeks of Gestation

(1) If enteral nutrition is possible, a dose of 800 IU/day (20 µg/day) of cholecalciferol is recommended from the first days of life, regardless of the feeding method, during the first month of life. The intake from a diet should be calculated from the second month of life. Calcifediol is not recommended.
(2) Supplementation should be monitored by serum 25(OH)D concentration assays, both during hospitalization (the first check-up after 4 weeks of supplementation) and/or followed up in the outpatient care.
(3) Total daily cholecalciferol dose of 1000 IU (25 µg/day) and higher may confer a risk of vitamin D overdose, especially in neonates with birth weight <1000 g.

#### 4.7.2. Neonates Born at 33–36 Weeks of Gestation

(1) A total of 400 IU/day (10 µg/day) of cholecalciferol from the first days of life, regardless of the feeding method, is recommended; calcifediol is not recommended.
(2) There is no need to control serum 25(OH)D concentrations routinely.
(3) Supplementation under the control of serum 25(OH)D concentration should be considered in neonates at a higher risk of vitamin D deficiency (parenteral nutrition lasting >2 weeks, ketoconazole therapy >2 weeks, anticonvulsant treatment, cholestasis, birth weight <1500 g).

## 5. Supplementation in Groups at Risk of Vitamin D Deficiency

(1) In patients at risk of vitamin D deficiency (Table 3), cholecalciferol or calcifediol supplementation should be implemented and followed up under the control of serum 25(OH)D concentrations, in order to achieve and maintain the optimal concentration of >30–50 ng/mL.
(2) If the assessment of serum 25(OH)D concentration is not possible in the risk groups, cholecalciferol dosing should be carried out according to the guidelines for the general population at the maximal doses for a given age group. Alternatively, calcifediol in a daily dose of 10 µg (oral solution) may be considered for preventive management.
(3) Overweight and obesity need a special attention as this condition usually requires a double dose of cholecalciferol in relation to the doses recommended for age-matched peers with normal body weight. In obese individuals, calcifediol in a daily dose of 10 µg (oral solution) may be considered as an alternative second choice of prevention scheme. Obesity in children and adolescents is defined as BMI >90th percentile for age and sex reference; obesity in adults and the elderly is defined as BMI >30 kg/m^2^.

**Table 3:** Risk groups for vitamin D deficiency or insufficiency according to a large body of published evidence and to Bleizgys [19].

Groups of Risk Factors	Examples: Diseases, Conditions, Lifestyle Features
Musculoskeletal disorders	Rickets, osteoporosis, osteopenia, “bone pains”, muscle pain, myophaty, myodystrophy, recurrent (“low energy”) bone fractures, recurrent falls, bone deformities
Endocrine and metabolic diseases/conditions	Diabetes mellitus (type 1 and II), metabolic syndrome, obesity, overweight, hypo- and hyperparathyroidism, hypo- and hyperthyroidism, hypocalcemia, calciuria, phosphatemia, hypo- and hyperphosphatasia, phosphaturia, dyslipidemias
Increased demand for physiological reasons	Childhood, adolescence, pregnancy, breastfeeding
Malabsorption syndromes	Pancreatic exocrine insufficiency (old age, pancreatitis, type II diabetes, etc.), inflammatory bowel disease (Crohn’s disease, ulcerative colitis), cystic fibrosis, celiac disease, bariatric surgery
Diseases of the liver and bile ducts	Hepatic insufficiency, chronic kidney disease (especially stages III–V), nephrotic syndrome
Respiratory diseases	Bronchiar asthma, chronic obstructive pulmonary disease
Infectious diseases	Tuberculosis, recurrent respiratory infections
Systemic connective tissue diseases	Rheumatoid arthritis, systematic lupus erythematosus, dermatomyositis, fribromyalgia
Skin diseases	Atopic dermatitis, psoriasis
Diseases of the nervous system	Multiple sclerosis, Parkinson’s disease, dementia, cerebral palsy, autism
Decreased production of vitamin D3 in the skin	Older age (especially >70 years) Active protection against sun exposure (sunscreens, etc.) Cultural features (usual full-body clothing) Rare outdoor activities (work and leisure predominantly indoors; living in a care home) Increased air pollution (living in a city) Winter season (at medium latitudes) Dark-skinned (especially Africans)
Nutritional features	Veganism and other types of vegetarianism Allergy to cow’s milk Low-fat diet Insufficient magnesium intake Insufficient calcium intake
Long-term use of drugs	Antiepileptic drugs (e.g., valporate, phenytoin); antiretroviral drugs; glucocorticoids; systemic antifungal drugs; rifampin; bile acid sequestrants (cholestyramine); lipase inhibitors (orlistat)
Malignant neoplasms	Colon cancer, lymphatic system and blood cancers, breast cancer, ovarian cancer, prostate cancer
Granulomatous diseases	Sarcoids, histoplasmosis, coccidiomycosis, berylliosis
Mental illnesses	Depression, schizophrenia, anorexia nervosa
Cardiovascular diseases	Arterial hypertension, ischemic heart disease, heart failure
Others	Chronic fatigue syndrome Inpatient treatment (especially in the resuscitation and intensive care unit) Awaiting organ transplantation and post-transplant

## 6. Supplementation in Specific Groups at Risk of Vitamin D Hypersensitivity

(1) Before starting supplementation, the risk of vitamin D hypersensitivity should be assessed if feasible (*SLC34A1* gene mutation, *CYP24A1* gene mutation, hypercalciuria, hypercalcemia, nephrolithiasis, nephrocalcinosis, or history of other types of vitamin D hypersensitivity in an individual or family members). Patients with chronic kidney disease, especially dialysis patients, kidney transplant recipients, are at the risk of inadequate activation of vitamin D by hydroxylation in position 1α by CYP27B1 and deactivation by CYP24A1, because both enzymes are mostly active in proximal tubules of the kidneys.
(2) In patients at risk of vitamin D hypersensitivity, supplementation should be supervised and carried out carefully, in an individual manner, always monitored with serum Ca, serum parathyroid hormone (PTH), serum 25(OH)D, serum 1,25(OH)_2_D and 24 hours calciuria (preferred over urinary Ca/creatinine ratio).
(3) Patients who suffer from chronic granuloma-forming disorders including sarcoidosis, tuberculosis, and chronic fungal infections and some patients with lymphoma have activated macrophages that produce 1,25(OH)_2_D in an unregulated fashion. These patients may require vitamin D treatment to raise their serum 25(OH)D to approximately 25 ng/mL [17,20]. The 25(OH)D concentrations need to be carefully monitored, because hypercalciuria and hypercalcemia are usually observed when the 25(OH)D is above 30 ng/mL [17,20].
(4) Patients with primary hyperparathyroidism and hypercalcemia are often vitamin D deficient. It is important to correct their vitamin D deficiency and maintain sufficiency. Most patients will not increase their serum calcium level, and serum PTH may even decrease. In patients with primary hyperparathyroidism serum 25(OH)D should be maintained >30 ng/mL. Supplementation with cholecalciferol should be cautious to prevent further increases in the serum or urinary calcium concentration [21].

## 7. Prophylactic and Treatment Recommendations Based on 25(OH)D Concentration Values

### 7.1. Assessment of Vitamin D Status and Diagnostic Criteria

It is recommended to measure both 25(OH)D_2_ and 25(OH)D_3_, giving a total 25(OH)D serum concentration as a measure of vitamin D status. The 25(OH)D TOTAL, with intraassay variation <5% and interassay variation <10%, should be subject to quality assurance by the certifying system DEQAS. The best assays provide total 25(OH)D concentration, excluding 3-epi-25(OH)D. The diagnostics thresholds defining concentrations of serum 25(OH)D in Poland are as follows:

(1) Concentrations ≤20 ng/ml (50 nmol/L) indicate vitamin D deficiency, a state that should be immediately treated medically with the use of therapeutic dosing.
(2) Concentrations of >20 ng/ml (50 nmol/L) <30 ng/ml (75 nmol/L) reflect a suboptimal vitamin D status that calls for a moderate increase of dosing.
(3) Concentrations of ≥30 ng/ml (75 nmol/L) up to 50 ng/ml (125 nmol/L) reflect adequate to optimal vitamin D status.
(4) Concentrations of >50 ng/ml (125 nmol/L) up to 100 ng/ml (250 nmol/L) indicate a high vitamin D supply.
(5) Concentrations higher than 100 ng/ml (250 nmol/L) reflect an increased risk for intoxication and need for a reduction/cessation of supplementation or treatment until obtaining target 25(OH)D concentration.

### 7.2. Principles of Supplementation and Treatment with Cholecalciferol and Calcifediol Based on Serum 25(OH)D Concentrations ≤20 ng/mL

(1) A 25(OH)D value of ≤20 ng/mL reflects an urgent need to start the medical intervention regimen.
(2) A single loading therapy with the use of a cholecalciferol dose of 100,000 IU and higher is not recommended in Poland.
(3) Cholecalciferol and calcifediol dosing for therapy of vitamin D deficiency should be based on serum 25(OH)D concentrations and previous prophylactic schemes.
(4) A daily and cumulative (weekly, biweekly, monthly) dosing regimen of therapy with the use of cholecalciferol in regards to obtaining and maintaining optimal 25(OH)D concentrations are complementary (1000 IU/d is equal to both 7000 IU/week and 30,000 IU/month, respectively), effective, and safe. Adjustment of the dosing of cholecalciferol regimen to the patient’s preferences and the therapy taken weekly or monthly can positively influence adherence. Caution is advised when using cholecalciferol inconsistently with the summary of product characteristics (SPCs).
(5) Daily (oral solution), weekly (soft capsules), biweekly (soft capsules), and monthly (soft capsules) dosing schemes of therapy with the use of calcifediol are safe but not equal in regards to an increase of 25(OH)D concentrations, therefore caution is advised when using calcifediol inconsistently with the summary of product characteristics (SPCs).

### 7.3. The Serum 25(OH)D Concentration >100 ng/mL—Increased Risk of Toxicity

(1) Vitamin D intoxication is defined as the condition in which serum 25(OH)D concentration >100 ng/mL is accompanied by hypercalcemia, hyperphosphatemia, hypercalciuria, and apparent PTH suppression.
(2) Therapy of vitamin D deficiency has to be stopped forthwith; calcemia and calciuria should be assessed, and serum 25(OH)D concentration should be monitored at 1-month intervals until a 25(OH)D concentration of ≤50 ng/mL is reached.
(3) In patients with clinical signs of vitamin D intoxication, appropriate evidence-based treatment should be immediately initiated.
(4) Verify if the previous therapy regimen was appropriate, and correct the management accordingly (intake, dosing, compliance, type of preparation).
(5) After reaching normocalcemia, normocalciuria, and 25(OH)D concentrations ≤50 ng/mL, prophylactic management or therapeutic intervention can be resumed after the exclusion of vitamin D hypersensitivity.

### 7.4. Serum 25(OH)D Concentrations >50–100 ng/mL—High Values

(1) Verify if the previous therapy regimen was appropriate, and correct the management accordingly (intake, dosing, compliance, type of preparation).

### 7.5. Serum 25(OH)D Concentrations >75–100 ng/mL

(1) Cholecalciferol or calcifediol therapy should be withheld for 1–2 months.
(2) In neonates, infants and toddlers, calcemia and calciuria should be assessed, vitamin D hypersensitivity should be excluded, and reevaluation of serum 25(OH)D concentration should be carried out.
(3) After 1–2 months or, in case of neonates, infants and toddlers, after reaching serum 25(OH)D concentration ≤50 ng/mL, a preventive dosing regimen may be restored.

### 7.6. Serum 25(OH)D Concentrations >50–75 ng/mL

(1) If cholecalciferol or calcifediol intake regimens were appropriate, it is recommended to reduce the current dosing or to suspend dosing for 1 month, and to consider assessment of 25(OH)D concentration within the consecutive 3-month period.

### 7.7. The Serum 25(OH)D Concentration ≥30–50 ng/mL—Optimal Values

(1) Continue previous management.

### 7.8. Suboptimal Serum 25(OH)D Concentration >20–30 ng/mL

(1) Verify if the previous therapy regimen was appropriate, and correct the management accordingly (intake, dosing, compliance, type of preparation).
(2) If the intake regimen was appropriate and the patient adhered to therapy correctly, it is recommended to increase the dosing of cholecalciferol, and to consider reassessment of serum 25(OH)D concentration in 6 months.
(3) In patients previously untreated, it is recommended to initiate the vitamin D supplementation using cholecalciferol at doses recommended for the general population.
(4) In case of inadequate response to supplementation, when previous use of cholecalciferol was ineffective and expected increase in serum 25(OH)D was not achieved, calcifediol in a daily (oral solution), biweekly (soft capsules), or monthly dosing (soft capsules) is recommended.

### 7.9. Serum 25(OH)D ≤20 ng/mL—Vitamin D Deficiency

(1) Verify if the previous therapy regimen was appropriate, and correct the management accordingly (intake, dosing, compliance, type of preparation).
(2) Therapeutic dose of cholecalciferol should be implemented immediately, based on age and body weight.
(3) Treatment of vitamin D deficiency should be continued for 1–3 months or until the serum 25(OH)D concentration of ≥30–50 ng/mL is achieved, then it is recommended to use consecutive maintenance dose i.e., a preventive dose recommended for the general population, in relation to age and body weight.
(4) In patients with skeletal symptoms, metabolic bone disease, and bone mineral disorders (bone deformations, bone pain, nonspecific musculoskeletal symptoms, fatigue syndrome, and history of fragility fractures), it is necessary to assess and monitor parameters of calcium-phosphate metabolism (Ca, PO_4_, ALP, PTH, urine Ca/creatinine ratio), and—if available—bone mineral density with the use of DXA.
(5) For some patients with chronic diseases (obesity, malabsorption syndromes, liver diseases, chronic inflammatory diseases) or that are taking medications that interfere with hepatic cytochrome P450 (i.e., glucocorticoids, anticonvulsants, anticancer or antiretroviral drugs) a quick restoration of vitamin D deficiency is needed. For those patients, the optional use of calcifediol in therapeutic biweekly or monthly doses of 266 μg (soft capsules) is reasonable, safe, and justified.
(6) After 1 to 3 months of cholecalciferol therapy, the reevaluation of serum 25(OH)D concentration should be performed.
(7) In patients receiving calcifediol in a daily dose of 10 μg (oral solution), or biweekly and a monthly dose of 266 μg (soft capsules) reevaluation of 25(OH)D concentration should be performed within 6–8 days, or 6–8 weeks, respectively.

### 7.10. Cholecalciferol Therapy

(1) From birth to 12 months of age: 2000 IU/day (50 µg/day); serum 25(OH)D concentration control assay no later than 4–6 weeks after.
(2) Age 1–10 years: 4000 IU/day (100 µg/day); serum 25(OH)D concentration control assay no later than 6–8 weeks after.
(3) Age 11–18 years: 4000 IU/day (100 µg/day) or 7000 IU/week (175 µg/week) or 10,000 IU/week (250 µg/week) or 20,000 IU taken biweekly (500 µg/biweekly) or 30,000 IU taken biweekly (750 µg/biweekly) or 30,000 IU/month (750 µg/month); serum 25(OH)D concentration control assay considered 8–12 weeks after, but not later, than up to 3 months after, depending on a dose of therapy.
(4) Age 19–64 years: 4000 IU/day (100 µg/day) or 7000 IU/week (175 µg/week) or 10,000 IU/week (250 µg/week) or 20,000 IU taken biweekly (500 µg/biweekly) or 30,000 IU taken biweekly (750 µg/biweekly) or 30,000 IU/month (750 µg/month); serum 25(OH)D concentration control assay considered 8–12 weeks after, but not later, than up to 3 months after, depending on a dose of therapy.
(5) Age 65–74 years: 4000 IU/day (100 µg/day) or 7000 IU/week (175 µg/week) or 10,000 IU/week (250 µg/week) or 20,000 IU taken biweekly (500 µg/biweekly) or 30,000 IU taken biweekly (750 µg/biweekly) or 30,000 IU/month (750 µg/month); serum 25(OH)D concentration control assay considered 8–12 weeks after, but not later, than up to 3 months after, depending on a dose of therapy.
(6) Age 75–89 years: 4000 IU/day (100 µg/day) or 7000 IU/week (175 µg/week) or 10,000 IU/week (250 µg/week) or 20,000 IU taken biweekly (500 µg/biweekly) or 30,000 IU taken biweekly (750 µg/biweekly) or 30,000 IU/month (750 µg/month); serum 25(OH)D concentration control assay considered 8–12 weeks after, but not later, than up to 3 months after, depending on a dose of therapy.
(7) Age 90 years and older: 4000 IU/day (100 µg/day) or 7000 IU/week (175 µg/week) or 10,000 IU/week (250 µg/week) or 20,000 IU taken biweekly (500 µg/biweekly) or 30,000 IU taken biweekly (750 µg/biweekly) or 30,000 IU/month (750 µg/month); serum 25(OH)D concentration control assay considered 8–12 weeks after, but not later, than up to 3 months after, depending on a dose of therapy.

### 7.11. Calcifediol Therapy

(1) From birth to 12 months of age: calcifediol is not recommended for this age group, unless other special considerations occur;
(2) 1–10 years: calcifediol is not recommended for this age group, unless other special considerations occur;
(3) 11–18 years: calcifediol in a dose of 10 µg daily (oral solution; for prevention) or 266 µg (soft capsules; for therapy) taken biweekly or monthly; the first serum 25(OH)D concentration control assay no later than 6–8 days after prevention with the use of 10 µg and 4–6 weeks after therapy with the use of 266 µg;
(4) 19–64 years: calcifediol in a dose of 10 µg daily (oral solution; for prevention) or 266 µg (soft capsules; for therapy) taken biweekly or monthly; the first serum 25(OH)D concentration control assay no later than 6–8 days after prevention with the use of 10 µg and 4–6 weeks after therapy with the use of 266 µg;
(5) 65–74 years: calcifediol in a dose of 10 µg daily (oral solution; for prevention) or 266 µg (soft capsules; for therapy) taken biweekly or monthly; the first serum 25(OH)D concentration control assay no later than 6–8 days after prevention with the use of 10 µg and 4–6 weeks after therapy with the use of 266 µg;
(6) 75–89 years: calcifediol in a dose of 10 µg daily (oral solution; for prevention) or 266 µg (soft capsules; for therapy) taken biweekly or monthly; the first serum 25(OH)D concentration control assay no later than 6–8 days after prevention with the use of 10 µg and 6–8 weeks after therapy with the use of 266 µg;
(7) 90 years and older: calcifediol in a dose of 10 µg daily (oral solution; for prevention) or 266 µg (soft capsules; for therapy) taken biweekly or monthly; the first serum 25(OH)D concentration control assay no later than 6–8 days after prevention with the use of 10 µg and 6–8 weeks after therapy with the use of 266 µg;

## 8. Basic Principles of Calcium Intake during Supplementation and Treatment with Vitamin D

(1) During the prevention and treatment of vitamin D deficiency, an appropriate dietary calcium intake should be assured, keeping in mind adequate hydration/rehydration.
(2) If adequate dietary calcium intake is not possible, calcium salts supplements are recommended, preferably in divided doses, which should be taken with meals, keeping in mind appropriate hydration.

## 9. Calcitriol and Active Analogues of Vitamin D

(1) Calcitriol and active analogues of vitamin D (e.g., alfacalcidol) should not be used to prevent vitamin D deficiency.
(2) Indications for treatment with these substances include conditions of impaired intrinsic vitamin D metabolism, such as renal failure or hypoparathyroidism.
(3) Significantly higher risks of overdose and intoxication necessitate monitoring of serum Ca, phosphate and alkaline phosphatase, and daily urinary calcium excretion.
(4) Attempts to assess 25(OH)D to monitor therapy with analogues is completely useless.

## 10. Discussion

### 10.1. Natural Vitamin D Sources in Brief

The incidence and mortality rates for cardiovascular and respiratory diseases are much higher in winter than in summer. As a result, all-cause mortality rates in winter are 25% higher than in summer in the U.S. [22,23]. Since the risk for cardiovascular disease and infectious diseases is inversely correlated with serum 25(OH)D concentrations, it would be very useful to understand why the concentrations change with season and how to maintain summertime concentrations in winter. The primary driver of seasonal changes is solar ultraviolet-B (UVB) exposure. Solar UVB comprises 3–5% of midday solar UV radiation in mid-latitudes near solar noon in summer. However, solar UVB drops to near zero in winter for about six months a year in Poland [24]. One can make vitamin D from solar UVB exposure only when one’s shadow is shorter than one’s height, whether by season or by time of day. Serum 25(OH)D concentrations in winter in the absence of solar UVB radiation are approximately 50–70% of summertime peak values [25,26,27]. The primary reason is that 25(OH)D is stored in muscles related to serum 25(OH)D concentrations as well as the amount of exercise, and is recirculated in the serum through the influence of PTH as needed, such as in winter when serum 25(OH)D concentrations fall [28,29,30]. Dietary sources of vitamin D such as meat and fish [26] also help maintain serum 25(OH)D concentrations in winter, but do not supply enough vitamin D to maintain summertime values in winter. Thus, raising vitamin D supplementation doses in winter must be encouraged. However, in practical terms, exposing 18% of the body to the sun without sunscreen in Poland for approximately 15–30 minutes and 30–45 minutes a day between 10 a.m. and 3 p.m. is likely to be adequate for fair-skinned children aged 4–10 years and adolescents, adults, and seniors, respectively. However, care should be taken in summer not to expose the skin to the point of erythema (reddening). Direct exposure to the sun is not recommended for infants and toddlers aged up to 4 years.

### 10.2. Role of Vitamin D for Human Health According to Selected RCTs

The classical role of vitamin D is regulation of absorption and metabolism of calcium and phosphorus. Most of these effects are controlled by modifying gene expression, however, non-genomic targets of a fast response to vitamin D were also described [31]. Nearly all cells in the body contain vitamin D receptor (VDR), which belongs to the family of nuclear receptors acting as transcription factor. When the hormonal metabolite of vitamin D, calcitriol, binds to the VDR, it can affect gene expression, either upregulating or downregulating them. A vitamin D supplementation study reported that for 400, 4000, or 10,000 IU/day vitamin D_3_ for 6 months, 162, 320, and 1289 genes were up- or down-regulated in their white blood cells, respectively [32]. This finding suggests that the increased risk of cardiovascular disease and mortality rates in winter is largely due to lower 25(OH)D concentrations in winter [23].

Serum 25(OH)D concentrations are inversely correlated with the risk of incidence and mortality rates for most diseases. The evidence regarding vitamin D and health outcomes comes from several types of studies. The medical system considers RCTs to provide the strongest evidence for the efficacy and adverse effects of pharmaceutical drugs. Unfortunately, RCTs with vitamin D supplementation designed and conducted in most of the 21st century have been based on the pharmaceutical drug model. Two assumptions of this model are that the trial is the only source of the studied agent and there is a linear dose-response relationship for the outcomes. In addition, growing knowledge about the health-promoting properties of vitamin D and widespread supplementation resulted in a general increase in 25(OH)D concentrations in the studied populations. This change is of course positive, but it is definitely harder to select control groups with low 25(OH)D concentration for RCTs or population-based studies. Examples of vitamin D RCTs that did not find the beneficial effects of vitamin D supplement include the Vitamin D and Omega-3 Trial (VITAL) regarding risk of cancer and cardiovascular disease [14] or the older reviews of RCTs [33] and a recent study on adult patients with thyroid disorders [34]. In VITAL, the mean 25(OH)D concentration for those in the treatment group was near 30 ng/mL, while in the thyroid study, the non-supplementing group had a mean 25(OH)D concentration of 26 ng/mL [14]. However, some studies in countries with very low mean 25(OH)D concentrations due to covering much of the skin and not getting enough vitamin D from diet or supplements can easily include many vitamin D-deficient participants. A good example is a study involving pregnant women in Iran [35]. The mean 25(OH)D concentration at baseline was 11 ng/mL and those treated with vitamin D supplementation to increase concentrations to above 20 ng/mL had a significantly reduced risk of gestational diabetes, preeclampsia, and preterm birth. Robert Heaney outlined the guidelines for nutrient trials, which would be appropriate for vitamin D [36]. The guidelines suggest that serum 25(OH)D concentrations be measured for all prospective participants, that those with low concentrations be included in the trial, that vitamin D doses be large enough to raise 25(OH)D concentrations high enough to significantly affect the health outcomes of interest, that achieved 25(OH)D concentrations be measured, and that the results should be based on serum 25(OH)D concentrations, not the presence or absence of vitamin D treatment. Since vitamin D RCTs have mostly not been properly designed, conducted, or analyzed, the effect of vitamin D has to be found from other types of studies [7].

### 10.3. Role of Vitamin D for Human Health According to Observational and Mendelian Randomization Studies in Brief

The most common other type of study is the observational study, generally based on serum 25(OH)D concentrations [7]. Observational studies are of three main types: prospective cohort studies, case-control studies, and cross-sectional studies. In the prospective cohort studies, participants are enrolled, blood samples taken, and the participants followed for times up to 10–20 years. Those who develop a health outcome of interest are paired with carefully selected controls who did not, and the risk ratios are calculated and adjusted for confounding factors.

Mendelian randomization studies examine the relationship between genetically determined serum 25(OH)D concentration and health outcomes in large databases. There are several steps involved in producing vitamin D in the skin and converting vitamin D to 25(OH)D concentration. Each step involves genes and the genes can have slightly different forms (alleles). The assumption is that, since individuals are randomized into study groups by the genetic variants they carry, bias due to confounding and reverse causation is avoided. It has recently been shown that analyzing the results for a large number of genetically determined 25(OH)D concentrations, thereby increasing the effect of very low 25(OH)D concentration, greatly improves the results as dose including data from ~300 000 participants, such as from the UK Biobank [6]. This methodology has already demonstrated causality for several health outcomes by that group including cardiovascular disease, dementia, and all-cause mortality rates, using data from the UK, Biobank as well as for hypertension, multiple sclerosis and type 2 diabetes mellitus by others that they cite.

To summarize, ecological studies have been used to find effects related to solar UVB doses, generally related to vitamin D production as done for cancer [37,38]. They have led to many other types of studies. Observational studies based on serum 25(OH)D concentrations of individuals and adjusted with respect to confounding risk-modifying factors have provided strong evidence that higher serum 25(OH)D concentrations are associated with reduced risk of many types of disease. Mendelian randomization studies are now able to demonstrate causality of vitamin D for many diseases [39], thereby replacing that role for RCTs, which generally have failed to do so [37]. Studies of mechanisms whereby vitamin D affects risk of disease also help confirm causality. Hill’s criteria for causality can be used with observational studies and mechanism to evaluate the evidence [40]. Hill’s criteria have been used in support of vitamin D in reducing risk of several types of disease including cancer [41] and cardiovascular disease [42].

### 10.4. Findings Regarding the Benefits of Higher 25(OH)D Concentrations

#### 10.4.1. Cardiometabolic Diseases

Since cardiometabolic diseases (diabetes, ischemic heart disease, and stroke) are the most important cause of death, they are the ones most important to understand the role of vitamin D. The first indication that vitamin D affected risk of cardiovascular disease (CVD) was likely the temporal ecological study by Robert Scragg in 1981, suggesting that the increased risk in winter was due to lower solar UVB doses and serum 25(OH)D concentrations [43]. It is now known that CVD mortality rates are about 25% higher in winter than in summer in mid-latitude countries and that lower serum 25(OH)D concentrations are the primary determinant. Observational studies of CVD incidence and mortality rates supported that hypothesis starting in 2008 [44]. A meta-analysis of prospective cohort studies regarding serum 25(OH)D and incidence and mortality from CVD was published in 2021 [45]. For the meta-analysis involving 28 studies, the relative risk for fatal CVD incidence declined from 1.0 at 8 ng/mL to 0.70 (96% CI, 0.65–0.75) at 25 ng/mL, i.e., a 30% reduction. Based on 10 studies, the risk of non-fatal CVD events was reduced by 20%, going from 8 ng/mL to 40 ng/mL (hazard ratio = 0.80 (95% CI, 0.70–0.95)).

A recently published article reported the risk of myocardial infarction as a function of serum 25(OH)D concentration for participants who had concentrations below 20 ng/mL at the start of the study [46]. The study ran for 20 years. For those who achieved 25(OH)D concentration from 21 and 29 ng/mL vs. <20 ng/mL, the hazard ratio was 1.14 (95% confidence interval (CI), 0.91–1.42), while for those who achieved >30 ng/mL vs. <20 ng/mL, the hazard ratio was 0.73 (95% CI, 0.55–0.96). It is noted that in the Vitamin D and Omega-3 Trial (VITAL), which enrolled over 25,000 participants and gave those in the vitamin D treatment arm 2000 IU/day vitamin D_3_, no effect of vitamin D supplementation was found for cardiovascular disease incidence or mortality [14]. However, the mean 25(OH)D concentration for those in the vitamin D treatment arm who supplied values, was 30 ng/mL. Thus, it could not be expected to find a beneficial effect when the whole data was analyzed. On the other hand, a stratified genetic-serum 25(OH)D concentration MR study did find a significant reduction in cardiovascular disease incidence [47].

#### 10.4.2. Diabetes Mellitus

Vitamin D also reduces the risk of both type 1 and type 2 diabetes mellitus. The mechanisms include influence on beta-cell function, insulin sensitivity, and systematic inflammation [48]. A meta-analysis of 8 RCTs with a total of 4896 prediabetic patients treated with vitamin D or placebo was reported in 2020 [49]. The pooled risk ratio for vitamin D treatment vs. placebo was 0.89 (95% CI, 0.80–0.99) while the pooled hazard ratio was 0.88 (95% CI, 0.78–0.99). For 1126 patients with BMI <25 kg/m^2^, the relative risk was 0.73 (95% CI, 0.57–0.92) while for 2514 patients with BMI >30 kg/m^2^, the relative risk was 0.95 (95% CI, 0.84–1.08). There are at least two reasons why patients with high BMI did not benefit as well as those with low BMI. One reason is that the vitamin D dose did not raise the serum 25(OH)D concentration as much as for those with low BMI. The second reason is that systemic inflammation increases with BMI [50]. Vitamin D supplementation has been found to reduce inflammation in non-obese patients [51] but not in obese patients [52].

Results from the secondary analysis of a vitamin D RCT involving prediabetic patients supplemented with 4000 IU/day vitamin D_3_ provides additional evidence of a beneficial effect of vitamin D. When the results were analyzed in terms of intention to treat, there was no significant difference in progression to type 2 diabetes mellitus. However, analysis of progression based on achieved 25(OH)D concentration for those supplemented with vitamin D, the hazard ratios for diabetes among participants treated with vitamin D who maintained intratrial 25(OH)D concentrations of 40–50 ng/ml (100–124 nmol/L) and ≥50 ng/mL (≥125 nmol/L) were 0.48 (0.29–0.80) and 0.29 (0.17–0.50), respectively, compared with those who maintained a 25(OH)D concentration of 20–30 ng/mL (50–74 nmol/L) [53].

#### 10.4.3. Cancer

The role of vitamin D in reducing risk of cancer was proposed in 1980 by the brothers Cedric and Frank Garland in a geographical ecological study of colon cancer mortality rates in the United States [54]. Colon cancer mortality rates were highest in the northeastern states, where annual solar radiation doses were lowest, and were lowest in the southwestern states, where doses were highest. Since then, numerous ecological studies have found inverse correlations between solar UVB doses, while many observational studies have found inverse correlations between serum 25(OH)D concentrations and cancer incidence, with reductions for high vs. low 25(OH)D concentrations of around 30% [38]. RCTs have only confirmed the role of vitamin D supplementation in reducing the risk of cancer mortality rates [55]. An RCT with 25,000 participants did show a reduced risk of all-cancer incidence rates for participants with BMI < 25 kg/m^2^ (HR =0.76 [95% CI, 0.63–0.90]) [14]. The shortcomings of that RCT included that the mean 25(OH)D concentration for participants in the vitamin D treatment arm was near 31 ng/mL, that they were given 2000 IU/d of cholecalciferol, that all participants were permitted to take an additional 600–800 IU/day of vitamin D depending on age, and that the results were not analyzed with respect to baseline and achieved 25(OH)D concentrations [14]. Another more recently published RCT conducted in the United States involved patients with metastatic colorectal cancer (CRC) in order to investigate if cholecalciferol in high doses added to standard chemotherapy improves the outcomes in patients with CRC [56]. The patients’ baseline 25(OH)D concentrations were close to 18 ng/mL and the study group was given 8000 IU/day of cholecalciferol for 2 weeks followed by 4000 IU/day for the rest of the study vs. 400 IU/day in controls [56]. The multivariable HR for progression-free survival or death was 0.64 (95% CI, 0–0.90) [56].

The mechanisms whereby vitamin D reduces the risk of cancer incidence include regulation of cellular differentiation, proliferation, and apoptosis (suicide), while those that reduce mortality also include reduced formation of blood vessels to supply tumors with nutrients and reduced metastasis into surrounding tissues [38].

#### 10.4.4. Infectious Diseases

Vitamin D has several mechanisms that help reduce the risk of viral and bacterial infections and progression to disease. One mechanism is that it induces the release of human cathelicidin, LL-37, a polypeptide with antimicrobial properties, making it an important component of the innate immune system [57,58,59]. It was proposed in 2020 that vitamin D could reduce the incidence and severity of COVID-19 [59,60]. Observational studies have found that higher 25(OH)D concentrations are associated with a reduced risk of COVID-19 [59,60,61]. A recent systematic review reported that vitamin D supplementation could significantly reduce the severity of COVID-19 in terms of reduced risk for intensive care, mechanical ventilation, and mortality [62].

A meta-analysis for double-blind RCTs for acute respiratory tract infection identified 1528 articles, of which 46 RCTs (75,541 participants) were eligible [8]. Data for the primary outcome were obtained for 48,488 participants (aged 0–95 years) in 43 studies. Protective effects were observed in a daily dosing regimen (OR = 0.78 [95% CI 0.65–0.94]; 19 studies), using 400–1000 IU/day (0.70 [0.55–0.89]; 10 studies), for 12 months or less (0.82 [0.72–0.93]; 29 studies), in young participants aged 1–16 years at enrolment (0.71 [0.57–0.90]; 15 studies) [8].

Respiratory syncytial virus (RSV)-associated acute respiratory infection causes substantial morbidity, leading to the hospitalization of 1 in every 56 healthy term–born infants in high-income settings [63]. An observational study conducted in the Netherlands on 256 neonates reported that those born with 25(OH)D concentrations <20 ng/mL compared with those with >30 ng/mL had an increased risk of developing severe lower respiratory tract infection RSV in the first year of life of 6.0 (95% CI, 1.6–24.9) [64].

Low vitamin D status also appeared as an important risk factor for sepsis incidence and mortality. In a meta-analysis involving 42 studies with 7434 children, 55% (95% CI, 49–61%) were vitamin D deficient [65]. Of the 889 children with sepsis from 18 studies, 64% (95% CI, 52–74%) were vitamin D deficient. The meta-analysis for mortality indicated that vitamin D deficiency increased the risk of death (OR = 1.81 [95% CI, 1.24–2.64]) [65].

#### 10.4.5. Autoimmune Diseases

Vitamin D also reduces risk of autoimmune diseases. The VITAL trial found that supplementing with 2000 IU/d vitamin D_3_ for 5.3 years significantly reduced the risk of autoimmune diseases [66]. The hazard ratio for vitamin D treatment compared to placebo was 0.78 (95% CI, 0.61–0.99). While results for individual autoimmune diseases were not significant due to low numbers of cases, the ones with reduced incidence were psoriasis, polymyalgia rheumatic disease, and rheumatoid arthritis. A recent review discussed the mechanisms whereby vitamin D reduces risk of autoimmune diseases [67]. The review also emphasized the preventive role of proper nutrition and maintaining sufficient vitamin D concentration in maternal blood during pregnancy, as well as in the early years of life.

#### 10.4.6. Pregnancy Outcomes

Vitamin D is very important during pregnancy and lactation. Higher 25(OH)D concentrations are associated with a reduced risk of Cesarean delivery, gestational diabetes, pre-eclampsia, and preterm delivery [68,69,70]. Optimal 25(OH)D concentrations during pregnancy are over 30 ng/mL (75 nmol/L).

A trial conducted in Iran with pregnant women shows how vitamin D RCTs should be conducted [35]. A total of 900 pregnant women were included at each of the 2 hospitals. The women at one hospital were not treated, while those at the other hospital, if vitamin D deficient, were treated with vitamin D. The mean values for many factors were well-matched at the time of enrollment including the 25(OH)D concentration, which was 11 ng/mL. A total of 800 of the women with vitamin D deficiency (<20 ng/mL) were divided into 2 groups; 10–20 ng/mL or <10 ng/mL. These groups were each further divided into 4 groups of 100 participants and supplemented with 50,000 IU/week for 12 weeks up to 300 000 IU each week for 6 weeks, followed by 50 000 IU/week until delivery. At delivery, serum 25(OH)D concentrations were 21 ng/mL (95% CI, 18–25 ng/mL) in the screened group vs. 11 ng/mL (7–16 ng/mL) in the unscreened group. All maternal outcomes were significantly reduced in the screened group, including pre-eclampsia, gestational diabetes, preterm delivery, and composite adverse pregnancy outcomes. The features that made this an excellent study include enrolling participants with vitamin D deficiency, giving sufficient vitamin D to raise serum 25(OH)D to where it reduced risk of adverse outcomes, measuring achieved 25(OH)D concentration, and not giving even small doses of vitamin D to the control group.

In a recent editorial, Hollis and Wagner discuss the importance of starting supplementation for women thinking of becoming pregnant with 4000 IU/day vitamin D_3_ [71]. They also point out that vitamin D RCTs should be based on serum 25(OH)D concentrations, not vitamin D dose, since RCTs based on dose are not appropriate since vitamin D per se is not the active agent, while serum 25(OH)D concentration is the best measure of the effect of vitamin D. They also point out that the reason why vitamin D is not getting more support for improving health is that it cannot be patented, and, thus, is not supported by health systems geared to use pharmaceutical drugs. 

## 11. Calcifediol—Introduction and Implementation into Clinical Practice

In the recent practice guidelines it was decided to include calcifediol as an alternative, second choice drug for both the prevention and treatment of vitamin D deficiency in special groups of patients. Calcifediol is 25(OH)D, the primary circulating vitamin D metabolite. It can be taken in Poland using an oral solution or soft capsules. There are some advantages to using calcifediol compared to cholecalciferol [72]. Oral calcifediol results in a faster increase in serum 25(OH)D than oral cholecalciferol, in hours instead of days. In addition, oral calcifediol has a higher rate of intestinal absorption, which may have important advantages in case of decreased intestinal absorption capacity due to a variety of diseases. In addition, oral calcifediol has a linear dose-response curve, irrespective of the baseline serum 25(OH)D concentration, whereas the rise in serum 25(OH)D is lower after oral cholecalciferol when the baseline serum 25(OH)D concentration is higher. Finally, an intermittent intake of calcifediol results in fairly stable serum 25(OH)D. The faster increase in serum 25(OH)D concentration can be an advantage when it is important to treat a vitamin D-responsive disease quickly, such as for COVID-19. A pilot RCT study in Spain reported significant reductions in COVID-19 severity with calcifediol treatment in 2020 [73]. A review published prior to the COVID-19 pandemic pointed out cholecalciferol, with calcifediol reserved for patients with liver failure or severe intestinal malabsorption syndromes [74]. In addition, calcifediol may be a good option for treating patients with inflammatory respiratory diseases, such as asthma, COPD, or COVID-19 since it can rapidly increase serum 25(OH)D concentrations to more quickly reduce the viability and proliferation of viruses and bacteria as well as reduce the production of pro-inflammatory cytokines that damage the surfaces of organs [75]. While accumulating evidence suggests that calcifediol may be an attractive alternative to “native” vitamin D, RCT data are still missing, but more data on this topic may be available in the future. At this stage however, we continue to recommend vitamin D_3_ (cholecalciferol) as a first-line substance to prevent and treat vitamin D deficiency and calcifediol as the second choice [76,77].

## 12. Conclusions

The vitamin D status of toddlers, children, adolescents, adults and seniors again needs to be paid special attention to. The present paper provides the clinical consensus on the prevention and treatment of vitamin D deficiency in Poland. Prophylaxis of vitamin D deficiency should be re-introduced for medical societies, medical professionals, and healthcare policymakers. It is strongly postulated to include practical guidelines on the prevention and treatment of vitamin D deficiency into every day practice.

## Figures and Tables

**Table 1:** Summary of previous vitamin D supplementation guidelines for Poland and Central Europe.

	2009 Polish Recommendations [16]	2013 Central European Recommendations [17]	2018 Polish Recommendations [18]
**Diagnostics thresholds defining vitamin D status on the basis of serum 25(OH)D concentration [ng/mL] †**			
Sufficiency	Children: 20–60 Adults: 30–80	30–50	30–50
Insufficiency	Not defined	20–30	20–30
Deficiency	<10	<20	10–20 deficiency <10 severe deficiency
Toxicity	Not defined	>100	>100
**Recommended daily doses of vitamin D—supplementation (daily doses in IU ‡)**			
Age			
0–6 months	400	400	400
6–12 months	400	400–600	400–600
2–18 years	400	600–1000	-
2–10 years	-	-	600–1000
11–18 years	-	-	800–2000
>18 years	800–1000	800–2000	800–2000
>75 years	-	-	2000–4000
Pregnancy and lactation	800–1000	1500–2000	2000
**Recommended daily doses of vitamin D—therapeutic doses for deficiency (daily doses in IU ‡)**			
Age			
0–1 month	1000	1000	
2–12 months	1000–3000	1000–3000	
0–12 months			2000
2–18 years	Up to 5000	3000–5000	
2–10 years			3000–6000
11–18 years			6000
>18 years	Up to 7000	7000–10,000 or 50,000/week	6000

† 1 ng/mL = 2.5 nmol/L; ‡ 40 IU = 1 μg.

## Data Availability

Not applicable.
